# Progress in the application of Enterococcus faecium in animal husbandry

**DOI:** 10.3389/fcimb.2023.1168189

**Published:** 2023-07-28

**Authors:** Zhi-lin Liu, Yun-jiao Chen, Qing-lei Meng, Xin Zhang, Xue-li Wang

**Affiliations:** Inner Mongolia Minzu University, College of Animal Science and Technology, Tongliao, Inner Mongolia, China

**Keywords:** Enterococcus faecium, biological characteristics, application, tolerance, virulence

## Abstract

As a probiotic, enterococcus faecium (E. faecium) has the characteristics of high temperature resistance, gastric acid resistance, bile salt resistance, etc. It can also effectively improve animal performance and immunity and improve the animal’s intestinal environment, so in recent years it has been more widely used in the livestock industry. However, due to the improper use of antibiotics and the growing environmental stress of strains, the drug resistance of enterococcus faecium has become more and more serious, and because some enterococcus faecium carry virulence genes, leading to the emergence of pathogenic strains, its safety issues have been widely concerned. This paper focuses on the biological characteristics of enterococcus faecium, the application of this bacterium in animal husbandry and the safety issues in its use, with a view to providing a reference for the application of enterococcus faecium in the development of animal husbandry.

## 1 Introduction

Lactic acid bacteria (LAB) are probiotics in the mammalian gut that regulate the balance of the host’s intestinal flora and improve host health (Liu et al., 2021). Enterococcus faecium, a Lactobacillus genus, is a commensal microorganism in the mammalian gut. Zhang Luyao et al. (Zhang et al., 2022) analysed the intestinal strain composition of F0 generation male ICR mice by sequencing the V3 to V4 regions of the 16SrRNA gene, and the results showed that the dominant flora in F0 at the genus level was the genus Lactobacillus, which accounted for 83.1% of the intestine. This reflects the possible proportion of enterococcus faecium in the mammalian gut. In addition to this, it produces antibacterial substances such as organic acids and bacteriocins.

E. Faecium has various excellent biological characteristics such as bile salt resistance, gastric acid resistance, heat resistance, fast growth, and strong adhesion (Kopit et al., 2014; Ju et al., 2018). As a probiotic, E. faecium is an important feed additive and is widely used in livestock production, with good prospects for feeding. Enterococcus faecium was listed as a safe strain for feed use by the FDA and AAFCO in 1984, and it was also listed in the Catalogue of Feed Additive Species (2013) issued by the Ministry of Agriculture in China in 2013 (Zhai, 2017). For example, E. Faecium SF68 is recommended as a probiotic supplement in the veterinary field (Krawczyk et al., 2021). Feng Baobao et al. (Feng et al., 2019) fed weaned piglets a feed containing 0.01% enterococcus faecium SF68. The results showed that the diarrhoea rate of piglets was significantly reduced, and the ratio of villus height to crypt depth of piglets’ duodenum, jejunum and ileum was significantly increased. The above results suggest that enterococcus faecium SF68 can reduce diarrhoea in weaned piglets by improving intestinal development and enhancing intestinal function. However, it is also considered as one of the opportunistic pathogens, and the national nosocomial infections surveillance (NNIS) has listed it as the second most common pathogen causing nosocomial infections (Holzapfel et al., 2018; Gorrie et al., 2019). Enterococcus faecium has been reported to be resistant to many antibiotics, with many of its antibiotic resistances present on plasmids and potentially transmitted by horizontal gene transfer (Arredondo-Alonso et al., 2020). Cho S et al. (Cho et al., 2022) used multiplex PCR to detect 21 plasmid replicon families of multidrug-resistant enterococcus faecium (696 strains) isolated from poultry carcass washings between 2004 and 2011. The results showed that 16% of enterococcus faecium (110/696) were positive for at least one plasmid replicon family. Li N et al. (Li et al., 2019) explored the intergeneric transfer of vancomycin resistance gene vanA during fermentation of soybean meal and in the digestive tract of growing pigs. The above studies confirm that drug-resistant enterococcus faecium possesses the ability to transfer plasmids to other enterococci.

The bacterium also carries virulence factors and virulence genes and has a high rate of infection and lethality in animals such as cattle, sheep and black bears. F. Dai et al. (Dai et al., 2018) injected enterococcus faecium isolated from black bear carcasses into mice and found that the mice showed symptoms such as lethargy, matted hair and loss of appetite, followed by death. Bai Weiqin et al. (Bai et al., 2021) isolated the suspected strain NEF1 from the brain tissue of diseased dead sheep in a sheep farm, and finally identified the strain as E. faecium by the combination of traditional bacterial isolation and identification and molecular biology. Therefore, the safety of feeding enterococcus faecium has attracted much attention. This article reviews the characteristics, application, and safety of this bacterium in animal husbandry production.

## 2 Biological characteristics of Enterococcus faecium

Enterococcus faecium is a gram-positive, facultative anaerobic bacteria. The colonies are round or oval, single or paired, and have no spore, no flagella, no motility and strong growth ability. Different strains of E. faecium are highly tolerant to temperature, pH and bile salts, and the bacterium can colonize and adhere to the intestinal tract of animals, with some inhibition of Staphylococcus aureus, Salmonella and Escherichia coli (Lee et al., 2019).

### 2.1 Heat resistance of Enterococcus faecium

Temperature can affect the activity of probiotics, and enterococcus faecium has good heat tolerance and will maintain its good activity when encountering high temperature conditions during feed processing. (Amaral et al., 2017). Zhou Bo et al. (Zhou et al., 2011) tested the inhibition activity of enterococcus faecium by placing the bacteria in a pressure steam sterilization pot at 121°C for 20 minutes and then performing an inhibition test using E. coli F41. The results showed that the inhibition diameter of the bacterium was only 12.50% lower compared to the non-heat treated samples, indicating that the bacterium is tolerant to high temperature environments. Zhang Ming et al. (Zhang et al., 2021) inoculated enterococcus faecium strain SC-Y112 in MRS liquid medium and incubated it at 30 °C for 24 h. The fermentation broth was put into a centrifuge at 6000 r/min and centrifuged at 4°C for 15 min, and the supernatant was extracted. Then, the supernatant was treated at 100 °C for 30 minutes, and the antibacterial activity of the fermentation broth was measured using a plate drilling method. The supernatant treated at room temperature was used as a control. The results showed that the inhibition ring of S. aureus in the control group was 11.1 ± 0.5 mm and 10.7 ± 0.4 mm in the 100°C treated S. aureus. This indicates that the effect of temperature on the fermentation broth of enterococcus faecium strain SC-Y112 was not significant.

In summary, enterococcus faecium has a greater advantage over other probiotics in terms of tolerance to high temperatures, which is one of the key reasons why it can be used as a feed additive for livestock.

### 2.2 Acid and bile salt resistance of Enterococcus faecium

Enterococcus faecium is well adapted to all environments and can survive in acidic and bile salt environments. The acid- and bile salt-tolerant properties of the bacterium allow it to continue to function in the organism during the animal’s digestion by resisting the action of gastric juice and bile. (Torres et al., 2018)

Bs S et al. (Bs et al., 2021) diluted enterococcus faecium BS5 in PBS (1:100) at different pHs (1.0, 2.0, 3.0, 4.0), followed by incubation on MRS Petri dishes at 37°C for 3h. The results showed that the survival rate of enterococcus faecium BS5 was 76% and 78% at pH 3.0 and 4.0 respectively, and around 25% at pH 1.0 and pH 2.0. Haijiayi et al. (Hai et al., 2021) showed that the survival rate of enterococcus faecium was about 20% when the pH value was 1~2. This indicates that enterococcus faecium can still grow stably in acidic conditions and has good acid tolerance. Mansour N et al. (Mansour et al., 2014) placed enterococcus faecium on MRS agar plates supplemented with bovine bile (0.3% and 0.7% w/v) for 3h at 37°C (no bile was set as control) and continued to incubate the bacteria on MRS agar plates for 48h at 37°C and found that the survival of the bacteria was about 83% in the presence of 0.3% bile and 68% in the presence of 0.7% bile. Shi Y et al. (Shi et al., 2020) added enterococcus faecium at 1% (v/v) to 0.1%, 0.2%, 0.3% and 0.6% (m/v) bile salt solutions and incubated them at 37°C for 3 hours. The experimental results showed that the survival rate of E. faecium in 0.1% bile salt for 3 h was close to 100%, in 0.2% and 0.3% bile salt for 3 h was around 90%, and in 0.6% bile salt for 3 h was close to 50%. All these indicated that enterococcus faecium had some tolerance to bile salts. The strain that survives after passing through the gastric juice encounters bile salts in the anterior part of the small intestine, so tolerance to bile salts is an important indicator that the strain can survive, grow and exert probiotic efficacy in the intestine (Luo et al., 2021), further indicating that the bacterium is well tolerated in the intestinal environment.

The above results show that enterococcus faecium, due to its good probiotic properties, can grow and multiply in the intestinal tract of animals, further producing lactic acid, bacteriocins and hydrogen peroxide to lower the intestinal pH and exert its antibacterial effect.

### 2.3 Adhesion and bacteriostasis of Enterococcus faecium

The adhesion and bacteriostasis of enterococcus faecium are

important conditions for its probiotic effect. E. faecium colonizes the host intestine and, together with other anaerobic bacteria, forms a biofilm flora that acts as a dominant protective agent, thereby reducing the adhesion of other pathogenic bacteria in the host intestine and contributing to the maintenance of intestinal health (Gong et al., 2016; Bai and Guo, 2021).

Ma Qingwen et al. (Ma et al., 2018) used PBS buffer to dilute Streptococcus equi DLR1-1-4, Streptococcus gallinolyticus DLR3-2-4, enterococcus faecium DLR3-3-3, enterococcus faecium DLR3-5-3, enterococcus faecium DLR4-7-4, and enterococcus faecium DLR6-12-2 by 10-fold gradient. The cells were resuspended in serum-free RPMI 1640, and then three strains with appropriate gradients were selected and added to the cells. After 48 hours of culture at 38°C, the number of bacteria adhered to each cell was calculated. Since Lactobacillus Rhamnose (LGG) has a strong adhesive ability, LGG is used as a comparison (Atarashi et al., 2015). The experimental results showed that the adhesion rates of six strains were 2.69%, 16.33%, 10.69%, 13.74%, 15.72% and 18.16% respectively, and the adhesion rate of LGG was 14.13%, of which the adhesion rate of enterococcus faecium DLR6-12-2 strain was the highest and higher than that of LGG, 18.16%. Hajikhani R et al. (Hajikhani et al., 2021) inoculated enterococcus faecium strains in MRS broth at 37 °C for 20 hours. The broth was centrifuged at 14000 r/min for 5 min to obtain the supernatant and five nutrient agar dishes containing different indicator bacteria (E. coli ATCC 35218, E. coli O157:H7, Salmonella enterica ATCC 13076, Salmonella typhimurium MU 80, Pseudomonas aeruginosa ATCC 27853) were punched. The supernatant was added to the wells and incubated at 37°C for 24 h before measuring the diameter of the inhibition zone. The results showed that E. faecium had inhibition rings of 7.0 mm, 4.8 mm, 4.8 mm, 5.2 mm and 0 mm against the five indicator bacteria, respectively. This suggests that E. faecium has an inhibitory effect on E. coli and Salmonella. Tian Fengsong et al. (Tian et al., 2017) added 240 μL of Lactobacillus (30 strains) supernatant to Oxford cups on prepared pathogenic plates, set equal amounts of sterile water as control group, and placed the plates with Oxford cups in an incubator at 37°C for 48 h. Four strains (Lactobacillus salivarius LWT1, enterococcus faecalis LWT2, enterococcus faecium LWT3, Streptococcus parinii LWT4) with strong bacteriostatic ability were screened out and the diameter of bacteriostatic ring was measured. The inhibition rings of the four strains against S. aureus, E. coli and Salmonella were LWT1: 20.97 ± 0.15, 12.30 ± 0.61, 12.83 ± 0.52; LWT2: 13.19 ± 0.72, 11.72 ± 0.40, 10.12 ± 0.30; LWT3: 12.31 ± 0.70, 10.65± 0.73, 10.78 ± 0.84; LWT4: 12.11 ± 0.27, 11.89 ± 1.03, 10.31 ± 0.77. It can be seen from the above results, enterococcus faecium has a certain inhibitory effect on Staphylococcus aureus, Escherichia coli and Salmonella. Compared with the other two bacteria, E Faecium has the most obvious inhibitory effect on Staphylococcus aureus.

The adherence of E. faecium allows it to colonise the intestinal tract of animals and the competitive adherence of the bacterium reduces the colonisation of other pathogenic bacteria in the intestinal tract, resulting in the suppression of pathogenic bacteria and thus contributing to the health of the intestinal tract and improving the efficiency of livestock farming.

## 3 Application of Enterococcus faecium in animal husbandry

### 3.1 Improvement of animal production performance

Enterococcus faecium in the animal’s intestine breaks down the carbohydrates in the feed to produce lactic acid, which is further degraded to acetic acid and propionic acid, etc. These organic acids improve the digestibility of proteins and carbohydrates as well as feed conversion and nutrient utilisation, which in turn improves animal performance (Ayalew et al., 2022).

Wang Yong et al. (Wang et al., 2013) measured the first and last weights of piglets at fasting and calculated the Average Daily Gain (ADG), Average Daily Feed Intake (ADFI) and Feed Conversion Ratio (F/G) of each piglet to determine the growth performance of piglets. The results showed that ADG of piglets with 500 mg/kg enterococcus faecium diet increased by 2.63%, ADFI decreased by 0.72%, and F/G decreased by 2.46%. Bai Tiantian et al. (Bai et al., 2022) found that adding 10mLE.faecium fermentation broth to sheep feed could increase the ADG of sheep from 71.24g/d to 81.33g/d. In addition, studies have shown that the addition of appropriate amounts of enterococcus faecium to feed can increase lactation in mid-lactation cows and improve milk quality. Hu Liangyu et al. (Hu et al., 2017) measured the milk volume and collected milk samples of Holstein cows fed with 60 g/d enterococcus faecium feed. The results showed an increase in lactation, milk protein content, non-fat milk solids content and dry matter lactation in the enterococcus faecium group compared to the basal diet group. They rose by 2.13kg, 0.05kg, 0.16kg and 0.09kg respectively. Some studies have found that enterococcus faecium is also of good use in poultry farming. Yu et al. (Yu et al., 2019) fed broilers three different concentrations of enterococcus faecium at 50mg/kg, 100mg/kg and 200mg/kg. It was found that compared to the blank control group, the broiler thigh meat redness increased by 0.88 and meat shear decreased by 4.71 in the low concentration (50mg/kg) E. faecium group; the broiler thigh meat redness increased by 2.16 and meat shear decreased by 3.20 in the medium concentration (100mg/kg) E. faecium group; the broiler thigh meat redness and meat shear increased by 2.71 and 2.93 respectively in the high concentration (200mg/kg) E. faecium group. Shear force increased by 2.71 and 2.93 respectively. Flesh colour is one of the important traits in meat quality, within a certain range, the higher the redness of the meat, the better the quality and freshness of the meat. The above results show that the bacterium can alter the quality of meat by changing the freshness and tenderness of broiler thigh meat, thus improving the growth performance of chickens. Cao Guangtian et al. (Cao et al., 2018) fed 360 7-day-old white feather broilers infected with Escherichia coli K88 (1 × 10^9^ CFU/mL) with diet supplemented with enterococcus faecium. The content of immunoglobulin in the serum of the infected broilers was determined by biotin double-antibody sandwich enzyme-linked immunosorbent assay (ELISA), and the broilers were weighed at the age of 7, 10, 14, 21 and 28 days to calculate their average daily weight gain. The results of the experiment showed an increase in immunoglobulin (IgA, IgM, IgG) content in serum and average daily weight gain in the enterococcus faecium group compared to the control group. IgA, IgM, and IgG levels increased by 4.52 ug/mL, 0.3 ug/mL, and 38.8 ug/mL, respectively. 7day old broiler ADG was reduced, by 1.3 g. Broiler flat ADG increased at 10, 14, 21 and 28 days of age, by 13.4 g, 43.9 g, 172.7 g and 229.7 g respectively. It can be seen that enterococcus faecium can improve the health status and growth rate of broilers infected with Escherichia coli.

### 3.2 Improvement of animal immunity

Enterococcus faecium as a feed additive can improve the immune level of animals, and it has the immunomodulatory function of inducing anti-inflammatory response and enhancing anti-pathogenic microbial infection (Makioka et al., 2018). Cui Bailei et al. (Cui et al., 2022) infused piglets with live bacteria containing non-pathogenic enterococcus faecium (1 × 10^8^ CFU/mL) skim milk solution. At the age of 7 and 21 days, 9 piglets close to the average body weight were randomly selected for slaughter. The jejunum and ileum segments were taken. The gene expression of tight junction protein, cytokine response and Toll-like receptor (TLR) were analysed by RNA extraction, cDNA preparation and real-time fluorescence quantitative PCR (RT-PCR) detection. The results showed that the relative expression of ZO-1 gene and interleukin-10 (IL-10) gene in the jejunum and ileum mucosa of 7-day-old piglets was on the rise, the relative expression of interleukin-8 (IL-8) gene was on the decrease and the relative expression of LR gene did not change significantly compared with the control group. The relative expression of IL-10 gene and transforming growth factor-β (TGF-β) in the jejunum and ileum of 21-day-old piglets was significantly increased, while the relative expression of IL-8 gene was significantly decreased, and the relative expression of ZO-1 gene and TLR gene were not significantly affected. These results indicate that the addition of enterococcus faecium promoted the expression of small intestinal tight junction protein gene, suppressed pro-inflammatory cytokines and increased the expression of anti-inflammatory cytokines, and stabilized the expression of TLR gene, thus improving the immunity level of piglets. Ding Shuang et al. (Ding et al., 2017) used ELISA kits to determine the immune function indicators (IgA, IgM, IgG) of piglets consuming E. faecium and found no significant change in IgA and IgM but a significant increase of 19.72% in IgG levels in the piglets’ serum compared to the basal diet group. The test showed that the addition of E. faecium to the feed increased the level of immunoglobulins in the serum of piglets. Xu Qiaoyun et a l. (Xu et al., 2018) added different doses (30 g/(d-head), 60 g/(d-head)) of E. faecium to the feed of Holstein cows in mid-lactation and took tail vein blood samples before morning feeding at d 0, 14 and 28 for the determination of immunoglobulins and other indicators. The results showed that there was an upward trend in the blood IgA content in the high-dose group at 28d, rising by 2.22 ng/ml, and there was no significant change in IgA and IgG content at other times. The levels of tumour necrosis factor in the high-dose group were significantly lower than those in the control and low-dose groups at the time. It indicates that enterococcus faecium can enhance the immunity of the body to some extent. Han Pengmin et al. (Han et al., 2022) took young pigeons added with enterococcus faecium (1.11 ×10^7^ CFU/mL) in the basic diet as the research object, determined immunoglobulin by ELISA and calculated their immune organ index according to the formula: immune organ index (%) = [immune organ weight (g)/live weight of young pigeons (g)] 100. The results revealed a non-significant difference in serum IgA levels, a significant increase in IgG levels by 16.1%, and an increase in thymus index, spleen index and bursa index by 0.02%, 0.04% and 0.03% respectively. These results show that the addition of enterococcus faecium to the diet can improve the immune level of suckling pigeons.

### 3.3 Improvement of the intestinal environment

**Figure 1:**
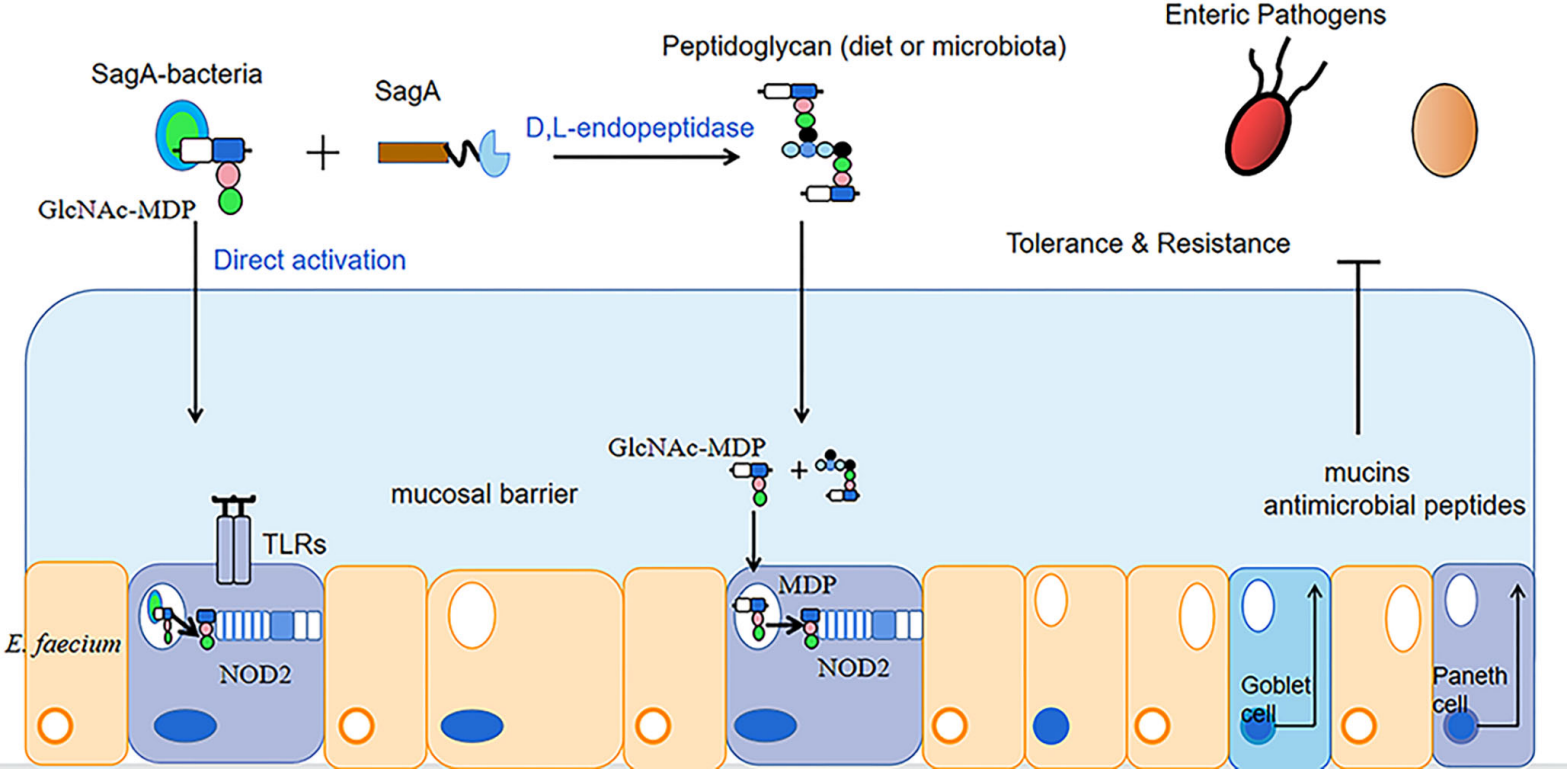
Mechanism of Enterococcus faecium enhancing intestinal function.

In addition to improving animal performance and immunity, enterococcus faecium can balance the intestinal flora and promote the growth of beneficial intestinal bacteria. Wang et al. collected excreta from AA broilers fed a basal diet and AA broilers fed a diet supplemented with E. faecium, dried and ashed them, and determined the phosphorus content of the ash in the ash samples by the vanadium-molybdate method, then AA broilers were electroshocked and slaughtered by hand within 5 min (Zhang et al., 2009; Liu et al., 2016; Wang et al., 2020), and intestinal mucosa was collected for the detection of intestinal flora. The results showed that the experimental group with the addition of enterococcus faecium showed a decrease in phosphorus excretion by 0.05 g/kg and a significant increase in the number of intestinal flora compared to the control group. The relative abundance of the genera Alistipes, Eubacterium, Rikenella and Ruminococcaceae was mainly increased. Dong Junsheng et al. (Dong et al., 2022) performed aseptic surgery on dairy goats continuously instilled with enterococcus faecium (1 × 10^9^ CFU/mL) for 4 d and took the goat jejunum and its contents for the detection of intestinal bacteria. The results revealed a significant increase in the number of lactic acid bacteria in the jejunum of the experimental group infused with enterococcus faecium, by 1 × 10^6^CUF/g, compared to the control group infused with an equal amount of saline. Kurbanaimu Kahman et al. (Kurbanaimu et al., 2022) used enterococcus faecium QH06 to gavage a portion of rats in a UC model, and after 24 h of fasting, whole sections of colonic tissue were taken for the detection of inflammatory factor expression levels and analysis of the Alpha diversity of the intestinal flora. The results showed that compared with the control group (uninstilled E. faecium QH06 group), the protein expression levels of IL-12 and IFN-γ in the E. faecium group decreased significantly, by 22 pg/mL and 26 pg/mL, respectively; the protein expression of IL-4 increased significantly, by 10 pg/mL. The Shannon, Ace and Chao indices all increased, by 0.97, 68.91 and 68.74, respectively, while the Simpson index decreased, by 0.21. The Simpson and Shannon indices reflect microbial community diversity and the Ace and chao indices reflect microbial community richness. It is evident that enterococcus faecium QH06 can improve the intestinal health of UC model rats. Peng X et al. (Peng et al., 2019) administered saline containing 1 × 10^8^ CFU/mL of enterococcus faecium NCIMB 10415 to piglets attacked with Escherichia coli K88 (saline administered under the same conditions was the control group) and found that compared with the control group, the relative abundance of Verrucomicrobiota in the group of enterococcus faecium after challenge showed an increasing trend, while the relative abundance of cholephilic bacteria showed a decreasing trend. The high abundance of Verrucomicrobiota is considered as a sign of intestinal health, because it has anti-inflammatory and intestinal barrier functions, which is conducive to the functioning of the colon barrier (Fujio-Vejar et al., 2017). High abundance of Bilophila wadsworthia can cause colitis and other intestinal inflammatory diseases. The potential mechanism of this bacteria causing intestinal inflammation is the production of sulphide to break the mucus barrier, so that bacteria can approach epithelial cells, and epithelial cells are damaged and inflamed (Devkota and Chang, 2015; Ijssennagger et al., 2016). Kim B et al. (Kim et al., 2019) found that peptide-glycan hydrolase (SagA) secreted by enterococcus faecium produced peptidoglycan in the presence of D, L-endonuclease, and that peptidoglycan degradation produced smaller cellular peptides that activated nucleotide-binding oligomeric domain protein 2 (NOD2) in mammalian cells. Furthermore, endogenous GlcNAc-MDP from enterococcus faecium can also directly stimulate and activate NOD2. Activated NOD2 regulates immune and barrier function in the gut through the production of mucin antimicrobial peptides to protect the host from intestinal pathogens, thereby improving the intestinal environment of the animal (Al Nabhani et al., 2017) (as shown in **Figure 1**).

The above studies show that enterococcus faecium is widely used in livestock farming and that its addition to feed can increase the diversity of the animal’s intestinal flora and regulate the ratio of intestinal flora to maintain intestinal health. As research on E. faecium continues, the safety of the bacterium is of increasing concern to the industry.

## 4 Safety of Enterococcus faecium in livestock and poultry industry

In most studies, most enterococcus faecium has been added to animal feeds as a probiotic to improve production performance. However, in recent years, studies have found that some E. faecium have become conditional pathogens of humans or animals, and I.D. Montioroni et al. (Montironi et al., 2020) isolated this bacterium from the milk of cows suffering from subclinical Mastitis. Due to the misuse of antibiotics in recent years, enterococcus faecium has developed some resistance to antibiotics (Soheili et al., 2014). In addition to this, the bacterium carries virulence genes and produces virulence factors. Dolka B et al. (Dolka et al., 2020) tested 17 strains of E. faecium isolated from cloacal samples of pigeons for virulence genes using PCR and showed that the 17 strains of E. faecium isolated carried the virulence genes gelE, asa1, efaA, ace, cylA, esp. Of these, cylA encodes a haemolysin (e. g. cytolysin) that causes haemocytolysis; asa1, gelE and esp genes encode biofilm-forming proteins that are involved in adherence to host cells, which in turn leads to infection of the organism (Bernardeau et al., 2008; Vandenesch et al., 2012; Zheng et al., 2018).

### 4.1 Enterococcus faecium carries virulence genes and virulence factors

E. faecium carries a variety of virulence genes (the main virulence genes are shown in **Table 1**) and virulence factors encoded by the virulence genes. The enzyme hyaluronidase encoded by Hyl helps pathogenic enterococcus faecium colonize host tissues by breaking down hyaluronic acid, a key component of connective tissue, thereby evading host immune defences and causing infection in the host (Rice et al., 2003; Hammad et al., 2014; Xiao et al., 2019).

**Table 1:** Names of major virulence genes carried by Enterococcus faecium.

Gene name	Gene expression products	Functions of gene expression products
efa A	Antigens of endocarditis	Infective endocarditis
bsh	Bile salt hydrolase	Regulate bile acid balance and affect lipid metabolism
esp	High molecular surface protein	Promote biofilm formation
acm	Glial protein adhesin	Promote cell wall anchored collagen adhesion
gel E	Gelatinase	Cause bacterial diffusion and participate in inflammatory reaction
asa 1	Surface aggregation protein	Mediate bacterial aggregation and plasmid transfer
cyl A	Cytolysin	Lytic cells
hyl	Hyaluronidase	Promote the spread of bacteria in connective tissue

Liu Yongan et al. (Liu et al., 2017) isolated strain XCP from dead sheep in a sheep farm in Sunan County, Gansu, and performed pathogenic bacteria isolation, Gram staining, biochemical tests, and 16SrRNA PCR amplification to identify the bacterium as enterococcus faecium. The strain of enterococcus faecium was also used for amplification of virulence genes (chyl, Asal, gelE, cylA, esp, acm) and pathogenicity assays in mice. The virulence gene amplification results showed that the strain carried the virulence genes Asal, cylA and Acm. The results of the pathogenicity test in mice showed that 10 mice that underwent intraperitoneal injection of enterococcus faecium solution (1×10^8^ CFU/mL) showed death and loss of appetite, and immediate dissection of the dead mice revealed enlarged livers and lungs, and all of them were able to isolate the inoculated bacteria. Montironi ID et al. (Montironi et al., 2020) used PCR to detect virulence genes in five E. faecium strains (EF-7A, EF-2, EF-3, EF-4, EF-5) isolated from the milk of cows with mastitis and found that the positivity rates for virulence genes detected from E. faecium strains were cyA 100%, efafm 100%, gelE 60%, agg 0% and esp 0%, respectively. At the same time, 100 μL, 1×10^8^ CFU/mL suspension of enterococcus faecium EF-7A strain was injected into the udder of lactating female rats and histopathological sections of mice were prepared. The results showed that the mammary gland tissue of the control dams was structurally intact, while strong infiltration of neutrophils (PMNs) was observed in the mammary glands of the inoculated dams within 48 hours, suggesting that the EF-7A strain of enterococcus faecium can infect the mammary glands of mice. The gelatinase encoded by the gel E gene, which was 60% positive for this experiment, is a zinc-containing protease that exerts its toxic effects by degrading collagen, fibrin and other important substances in the organism. For example, Gel E cleaves the allergenic toxin complement C5a, and hydrolysis of complement protein C5a prevents neutrophil migration to the site of infection, while the protein also regulates cytokine production and participates in the inflammatory response of the body (Thurlow et al., 2010). Wu Lixian et al. (Wu et al., 2009) prepared a mouse intraperitoneal injection with a knockout hy l gene strain of enterococcus faecium (named ∗hy l) bacterial solution and 30% sterile rat faecal extract (SRFE) in a ratio of 1:9 and injected it (1 ml) into the intraperitoneal cavity of mice. The results showed that the LD50 of the control group with equal conditions without hy l gene knockdown was 1.3×10^8^cfu and the LD50 of ∗hy l was 9.2×10^8^cfu, and the LD50 was increased approximately 7-fold after hy l gene knockdown. The survival rate of mice with peritonitis in the ∗hy l group was also significantly increased compared to the control group. This indicates that the virulence of the hy l knockout strain of ∗hy l underwent a significant decrease. This suggests that the hy l gene may be a more important factor in the pathogenesis of E. faecium. In addition to this, the CC17 complex of enterococcus faecium can generate greater viability through the acquisition of exogenous genes, thereby causing serious harm to the organism (Yang et al., 2017). Enterococcus faecium can carry a variety of virulence genes at the same time, leading to a gradual increase in the pathogenicity of the bacterium to the organism, thus posing a serious risk to the livestock industry.

### 4.2 Drug resistance of Enterococcus faecium

Currently, E. faecium is resistant to several antibiotics, which makes the treatment of animal diseases caused by the bacterium (bacteraemia, endocarditis, etc.) more difficult, thus posing a threat to the health of the animal organism (Gao et al., 2019).

**Figure 2:**
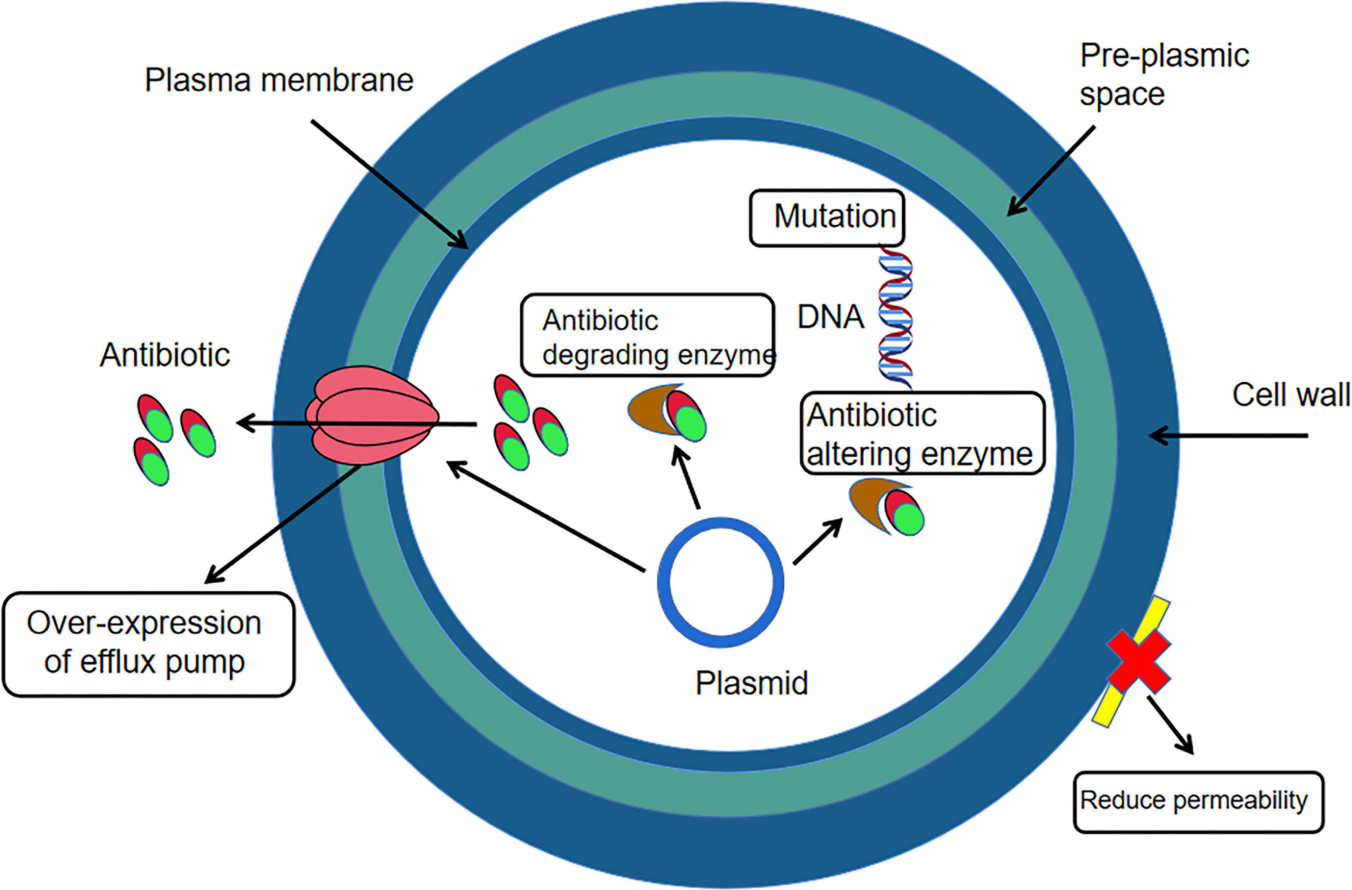
Mechanism of drug resistance in Gram-positive bacteria.

Yuksekdag Z et al. (Yuksekdag et al., 2021) used the paper diffusion method to determine the susceptibility of antibiotics such as gentamicin, polymyxin-B, ofloxacin and rifampicin to 17 strains of enterococcus faecium in white cheese samples. The results found that enterococcus faecium was 100%, 100%, 76% and 53% resistant to the four antibiotics respectively. Li Jia et al. (Li et al., 2017) tested the susceptibility of enterococcus faecium to ampicillin, erythromycin, ciprofloxacin, gentamicin and tetracycline using the VITEK2 Compact fully automated bacteriological analyser and found that the resistance rate of the bacteria to erythromycin, ciprofloxacin and gentamicin was above 85%, with the highest resistance rate of 93.1% to erythromycin. El-Zamkan MA et al. (El-Zamkan and Mohamed, 2021) inoculated a suspension of enterococcus faecium at a concentration of 10^5^ CFU/mL on Mueller-Hinton agar plates and placed different drug-sensitive tablets (penicillin, zocillin, erythromycin, tetracycline, linezolid, furantoin) on the surface of the medium separately and then incubated at 37˚C for 24 h. The results showed that enterococcus faecium had the highest resistance to zocillin at 100%, 66.7% to erythromycin and linezolid, 58.3% to penicillin and furantoin and the lowest resistance to tetracycline at 0%. Linezolid is commonly used for infections caused by drug-resistant gram-positive bacteria and is a rare medical drug, but the misuse of antibiotics in the veterinary field has necessitated testing for resistance to linezolid (Qin et al., 2022). Jahansepas et al. (Jahansepas et al., 2018) determined the susceptibility of antibiotics such as ciprofloxacin, ampicillin, penicillin and erythromycin to enterococcus faecium based on the disc diffusion method and showed that the resistance rates of E. faecium to ciprofloxacin, gentamicin, penicillin and erythromycin were 91.4%, 85.7%, 88.6% and 97.1%, respectively. In addition to this, studies in recent years have found that enterococcus faecium is also resistant to vancomycin (vancomycin is not used in animal husbandry, but due to the irrational use and misuse of antibiotics, the veterinary field should always be aware of changes in vancomycin resistance in enterococcus faecium and thus be prepared for possible situations in advance.) However, vancomycin is one of the more effective drugs available for the treatment of enterococcal infections, which makes this bacterium a serious threat to public health and industrial and agricultural production (Bonten et al., 2001). E. faecium is a Gram-positive bacterium and the mechanism of resistance in Gram-positive bacteria (**Figure 2**) can occur through two main strategies: enzymatic degradation of antibiotics through the production of β-lactamases, or through the acquisition of exogenous DNA or alteration of the natural PBP gene to reduce the affinity and susceptibility of its target penicillin-binding protein (PBP) (Karaman et al., 2020). Therefore, the mechanisms of drug resistance of this organism should be further investigated, and thus facilitate the development of rational clinical treatment protocols and prevention strategies for the infection and transmission of drug-resistant bacteria.

## 5 Conclusion and expectation

Enterococcus faecium stands out among livestock feed additives due to its high quality biological properties (resistance to heat, acid, bile salts, adhesion and bacterial inhibition) and its probiotic properties. Non-pathogenic enterococcus faecium strain, as a probiotic additive, can improve the production performance and immunity of animals, and can also improve the Internal environment of animals’ intestines, so it has a good application prospect in animal husbandry production. However, due to increasing drug resistance, the safe use of enterococcus faecium is now a problem that needs to be addressed. With the intensive research on enterococcus faecium, it has been confirmed that the bacterium is conditionally pathogenic and potentially pathogenic, and that the pathogenicity of the bacterium is closely linked to its virulence genes. Therefore, screening for virulence genes and virulence factors should be strengthened before using E. faecium to ensure its safety. The resistance of enterococcus faecium to different antibiotics is based on different mechanisms. It is necessary to conduct an in-depth study on the resistance mechanisms of enterococcus faecium to further propose effective measures for the prevention and treatment of diseases caused by enterococcus faecium.

## Author contributions

All authors listed have made a substantial, direct, and intellectual contribution to the work and approved it for publication.

## References

[B1] Al Nabhani Z. Dietrich G. Hugot J. P. Barreau F. (2017). Nod2: The intestinal gate keeper. PloS Pathog. 13 (3), e1006177. doi: 10.1371/journal.ppat.1006177 28253332 PMC5333895

[B2] Amaral D. M. F. Silva L. F. Casarotti S. N. Nascimento L. C. S. Penna A. L. B. (2017). Enterococcus faecium and Enterococcus durans isolated from cheese: Survival in the presence of medications under simulated gastrointestinal conditions and adhesion properties. J. Dairy Sci. 100 (2), 933–949. doi: 10.3168/jds.2016-11513 27988121

[B3] Arredondo-Alonso S. Top J. McNally A. Puranen S. Pesonen M. Pensar J.. (2020). Plasmids shaped the recent emergence of the major nosocomial pathogen enterococcus faecium. mBio. 11 (1), e03284–e03219. doi: 10.1128/mBio.03284-19 32047136 PMC7018651

[B4] Atarashi K. Tanoue T. Ando M. Kamada N. Nagano Y. Narushima S.. (2015). Th17 cell induction by adhesion of microbes to intestinal epithelial cells. Cell. 163 (2), 367. doi: 10.1016/j.cell.2015.08.058 26411289 PMC4765954

[B5] Ayalew H. Zhang H. Wang J. Wu S. Qiu K. Qi G.. (2022). Potential feed additives as antibiotic alternatives in broiler production. Front. Vet. Sci. 9. doi: 10.3389/fvets.2022.916473 PMC9247512 35782570

[B6] Bai T. T. Cui H. R. Zhao L. B. Wang Y. Guo X. F. Zhou X. L. (2022). Effects of Adding Enterococcus faecalis, Bacillus subtilis and Their Compound Bacteria on Growth Performance, Nutrient Apparent Digestibility, Rumen Fermentation Indexes and Rumen Microflora of Sheep. J. Anim. Nutr. 34 (08), 5190–5205. doi: 10.3969/j.issn.1006-267x.2022.08.042

[B7] Bai T. Guo X. (2021). The characteristics of Enterococcus faecium and its application in animal production. Chin. J. Anim. Husbandry. 57 (02), 16–20. doi: 10.19556/j.0258-7033.20200329-03

[B8] Bai W. Q. Li H. Meng K. Zi Z. F. Li J. Zhao L. J.. (2021). Isolation and identification of 1 strain of sheep pathogenic enterococcus faecium. Chin. Anim. husbandry veterinary Med. 48 (10), 3889–3895. doi: 10.16431/j.cnki.1671-7236.2021.10.042

[B9] Bernardeau M. Vernoux J. P. Henri-Dubernet S. Guéguen M. (2008). Safety assessment of dairy microorganisms: the Lactobacillus genus. Int. J. Food Microbiol. 126 (3), 278–285. doi: 10.1016/j.ijfoodmicro.2007.08.015 17889388

[B10] Bonten M. J. Willems R. Weinstein R. A. (2001). Vancomycin-resistant enterococci: why are they here, and where do they come from? Lancet Infect. Dis. 1 (5), 314–325. doi: 10.1016/S1473-3099(01)00145-1 11871804

[B11] Bs S. Thankappan B. Mahendran R. Muthusamy G. Femil Selta D. R. Angayarkanni J. (2021). Evaluation of GABA production and probiotic activities of enterococcus faecium BS5. Probiotics Antimicrob. Proteins. 13 (4), 993–1004. doi: 10.1007/s12602-021-09759-7 33689135

[B12] Cao G. T. Dai B. Zhang L. L. Zen X. F. Yang C. M. (2018). Effects of Enterococcus faecium on the Growth Performance,Serum Biochemical Parameters and the Structure of Cecal Microflora in the E. coli-challenged Broilers. J. Anim. Husbandry Veterinary Med. 49 (05), 962–970. doi: 10.11843/j.issn.0366-6964.2018.05.011

[B13] Cho S. McMillan E. A. Barrett J. B. Hiott L. M. Woodley T. A. House S. L.. (2022). Distribution and Transfer of Plasmid Replicon Families among Multidrug-Resistant Enterococcus faecalis and Enterococcus faecium from Poultry. Microorganisms. 10 (6), 1244. doi: 10.3390/microorganisms10061244 35744761 PMC9228330

[B14] Cui B. L. Wang L. Guo Q. P. Huang Y. Hu S. L. (2022). Effects of Enterococcus faecium on the expression of tight junction protein, cytokine response and Toll-like receptor gene in piglets' small intestine. J. Anim. Nutr. [Preprint]. . 34 (12), 7616–7627.

[B15] Dai F. Xiang X. Duan G. Duan B. Xiao X. Chang H. (2018). Pathogenicity characteristics of Enterococcus faecium from diseased black bears. Iran J. Vet. Res. 19 (2), 82–86. 30046317 PMC6056144

[B16] Devkota S. Chang E. B. (2015). Interactions between diet, bile acid metabolism, gut microbiota, and inflammatory bowel diseases. Dig Dis. 33 (3), 351–356. doi: 10.1159/000371687 26045269 PMC4477270

[B17] Ding S. Guo C. H. Zhang Z. F. Bo X. Wei J. Zhang M.. (2017). Enterococcus faecium B producing lactobacillus_ (13) Effects on growth performance, nutrient digestibility, serum immune index and fecal microbial flora of weaned piglets. J. Anim. Husbandry Veterinary Med. 48 (10), 1902–1911.

[B18] Dolka B. Czopowicz M. Chrobak-Chmiel D. Ledwoń A. Szeleszczuk P. (2020). Prevalence, antibiotic susceptibility and virulence factors of Enterococcus species in racing pigeons (Columba livia f. domestica). BMC Vet. Res. 16 (1), 7. doi: 10.1186/s12917-019-2200-6 31910839 PMC6947970

[B19] Dong J. S. Jiang Y. S. Li Z. X. Cui L. Y. Wang H. Li J. J. (2022). Therapeutic effect of Enterococcus faecium on colitis in goats. Chin. Veterinary Sci. 53 (02), 262–268. doi: 10.16656/j.issn.1673-4696.2023.0014

[B20] El-Zamkan M. A. Mohamed H. M. A. (2021). Antimicrobial resistance, virulence genes and biofilm formation in Enterococcus species isolated from milk of sheep and goat with subclinical mastitis. PloS One 16 (11), e0259584. doi: 10.1371/journal.pone.0259584 34780540 PMC8592430

[B21] Feng B. B. Fang W. Zhao G. Q. Huo Y. J. (2019). Effect of Enterococcus faecium SF68 on diarrhea rate, intestinal development and histomorphology in weaned piglets[J]. J. Anim. Nutr. 31 (02), 940–948. doi: 10.3969/j.issn.1006-267x.2019.02.051

[B22] Fujio-Vejar S. Vasquez Y. Morales P. Magne F. Vera-Wolf P. Ugalde J. A.. (2017). The gut microbiota of healthy chilean subjects reveals a high abundance of the phylum verrucomicrobia. Front. Microbiol. 8 (1). doi: 10.3389/fmicb.2017.01221 PMC5491548 28713349

[B23] Gao Y. Huang J. H. Chen L. Wang M. L. Sun J. J. Wang L. P.. (2019). Antimicrobial susceptibility and amphenicols resistance genes in Enterococcus faecium isolated from swine in the northern of Jiangsu Province. J. Nanjing Agric. University. 42 (02), 322–327. doi: 10.7685/jnau.201806024

[B24] Gong H. Wang H. X. Ma Z. T. Wang C. Y. Feng Q. Ma Y. Y. (2016). Study on adhesion of lactic acid bacteria and biofilm, hydrophobicity and self-agglutination characteristics. Chin. J. Microbiol. 28 (09), 1026–1028+1033. doi: 10.13381/j.cnki.cjm.201609008

[B25] Gorrie C. Higgs C. Carter G. Stinear T. P. Howden B. (2019). Genomics of vancomycin-resistant Enterococcus faecium. Microb. Genom. 5 (7), e000283. doi: 10.1099/mgen.0.000283 31329096 PMC6700659

[B26] Hai J. Y. Zheng S. S. Hou J. P. Long X. Wang W. Zhu L. L. (2021). Isolation, identification and biological characteristics of beneficial bacteria from the intestines of alpine vultures [J]. Adv. Anim. Med. 42 (12), 138–144. doi: 10.3969/j.issn.1007-5038.2021.12.026

[B27] Hajikhani R. Onal Darilmaz D. Yuksekdag Z. N. Beyatli Y. (2021). Assessment of some metabolic activities and potential probiotic properties of eight enterococcus bacteria isolated from white cheese microbiota. Antonie Van Leeuwenhoek. 114 (8), 1259–1274. doi: 10.1007/s10482-021-01599-3 34086120

[B28] Hammad A. M. Shimamoto T. Shimamoto T. (2014). Genetic characterization of antibiotic resistance and virulence factors in Enterococcus spp. from Japanese retail ready-to-eat raw fish. Food Microbiol. 38, 62–66. doi: 10.1016/j.fm.2013.08.010 24290627

[B29] Han P. M. Zhang R. Bian S. X. Li Y. L. Ni A. X. Zhang Y. M.. (2022). Effects of Enterococcus faecium and Bacillus subtilis on growth performance, slaughter performance and immune function of young pigeons. J. Anim. Husbandry Veterinary Med. [Preprint] . 53 (11), 3880–3891.

[B30] Holzapfel W. Arini A. Aeschbacher M. Coppolecchia R. Pot B. (2018). Enterococcus faecium SF68 as a model for efficacy and safety evaluation of pharmaceutical probiotics. Beneficial Microbes 9(3), 375–388. doi: 10.3920/BM2017.0148 29633645

[B31] Hu L. Y. Yuan X. Wang M. Z. Huo Y. J. Luo Y. H. (2017). Effects of Enterococcus faecium preparation on lactation performance and somatic cell classification of dairy cows in mid-lactation. Feed industry. 38 (09), 55–60. doi: 10.13302/j.cnki.fi.2017.09.011

[B32] Ijssennagger N. van der Meer R. van Mil S. W. (2016). Sulfide as a mucus barrierbreaker in inflammatory bowel disease? Trends Mol. Med. 22 (3), 190–199. doi: 10.1016/j.molmed.2016.01.00 26852376

[B33] Jahansepas A. Aghazadeh M. Rezaee M. A. Hasani A. Sharifi Y. Aghazadeh T.. (2018). Occurrence of Enterococcus faecalis and Enterococcus faecium in Various Clinical Infections: Detection of Their Drug Resistance and Virulence Determinants. Microb. Drug Resist. 24 (1), 76–82. doi: 10.1089/mdr.2017.0049 28525287

[B34] Ju A. Q. Gu. W. Zhang H. P. Qu L. Zhang D. X. Chen L.. (2018). Effects of Enterococcus faecium on non-specific immune enzyme activity and disease resistance of carp [J]. Chin. veterinary science. 48 (02), 228–233. doi: 10.16656/j.issn.1673-4696.2018.0035

[B35] Karaman R. Jubeh B. Breijyeh Z. (2020). Resistance of gram-positive bacteria to current antibacterial agents and overcoming approaches. Molecules. 25 (12), 2888. doi: 10.3390/molecules25122888 32586045 PMC7356343

[B36] Kim B. Wang Y. C. Hespen C. W. Espinosa J. Salje J. Rangan K. J.. (2019). Enterococcus faecium secreted antigen A generates muropeptides to enhance host immunity and limit bacterial pathogenesis. Elife. 8, e45343. doi: 10.7554/eLife.45343 30969170 PMC6483599

[B37] Kopit L. M. Kim E. B. Siezen R. J. Harris L. J. Marco M. L. (2014). Safety of the surrogate microorganism enterococcus faecium NRRL b-2354 for use in thermal process validation. Appl. Environ. Microbiol. 80 (6), 899–909. doi: 10.1128/AEM.03859-13 PMC3957640 24413604

[B38] Krawczyk B. Wityk P. Gałęcka M. Michalik M. (2021). The many faces of enterococcus spp.-commensal, probiotic and opportunistic pathogen. Microorganisms 9 (9), 1900. doi: 10.3390/microorganisms9091900 34576796 PMC8470767

[B39] Kuerbanaimu K. Zhao J. Mukaidaisi A. Wang H. Zhu J. Pan W.. (2022). Enterococcus faecalis QH06 attenuates colonic mucosal damage in rats with ulcerative colitis. Journal of Southern Medical University 42 (07), 976–987. doi: 10.12122/j.issn.1673-4254.2022.07.03 35869759 PMC9308865

[B40] Lee T. Pang S. Abraham S. Coombs G. W. (2019). Antimicrobial-resistant CC17 Enterococcus faecium: The past, the present and the future. J. Glob Antimicrob. Resist. 16, 36–47. doi: 10.1016/j.jgar.2018.08.016 30149193

[B41] Li J. Liu G. Y. Hu L. L. Kang H. Q. Deng L. H. Gu B.. (2017). Distribution and drug resistance analysis of clinical isolates of Enterococcus faecalis and Enterococcus faecalis from 2011 to 2015. J. Nanjing Med. Univ. (Natural Sci. Edition). 37 (10), 1353–1356. doi: 10.7655/NYDXBNS20171032

[B42] Li N. Yu H. Liu H. Wang Y. Zhou J. Ma X.. (2019). Horizontal transfer of vanA between probiotic Enterococcus faecium and Enterococcus faecalis in fermented soybean meal and in digestive tract of growing pigs. J. Anim. Sci. Biotechnol. 10, 36. doi: 10.1186/s40104-019-0341-x 31044075 PMC6460829

[B43] Liu Y. A. Li X. R. Zhou J. H. Ma L. N. Liu Y. S. (2017). Isolation, identification and drug resistance analysis of a sheep derived pathogenic Enterococcus [J]. China Anim. Husbandry Veterinary J. 44 (02), 530–537. doi: 10.16431/j.cnki.1671-7236.2017.02.032

[B44] Liu S. B. Liao X. D. Lu L. Li S. F. Wang L. Zhang L. Y.. (2016). Dietary non-phytate phosphorus requirement of broilers fed a conventional corn-soybean meal diet from 1 to 21 d of age. Poult. Sci. 96 (1), 151–159. doi: 10.3382/ps/pew212 27486251

[B45] Liu Z. Xu C. Tian R. Wang W. Ma J. Gu L.. (2021). Screening beneficial bacteriostatic lactic acid bacteria in the intestine and studies of bacteriostatic substances. J. Zhejiang Univ Sci. B. 22 (7), 533–547. doi: 10.1631/jzusB2000602 34269007 PMC8284090

[B46] Luo Q. Zhang M. Liu Q. Luo P. (2021). Evaluation of probiotics and safety of Enterococcus faecium SC-Y112 producing bacteriocin in *vitro* . Food Science. 42 (11), 154–160. doi: 10.7506/spkx1002-6630-20200602-025

[B47] Ma Q. W. Wang X. L. Wang Y. J. Huang A. X. (2018). Isolation and identification of lactic acid bacteria in goat milk cake and preliminary screening of excellent lactic acid bacteria. China dairy industry. 46 (03), 8–12. doi: 10.3969/j.issn.1001-2230.2018.03.002

[B48] Makioka Y. Tsukahara T. Ijichi T. Inoue R. (2018). Oral supplementation of Bifidobacterium longum strain BR-108 alters cecal microbiota by stimulating gut immune system in mice irrespectively of viability. Biosci. Biotechnol. Biochem. 82 (7), 1180–1187. doi: 10.1080/09168451.2018.1451738 29557273

[B49] Mansour N. M. Heine H. Abdou S. M. Shenana M. E. Zakaria M. K. El-Diwany A. (2014). Isolation of Enterococcus faecium NM113, Enterococcus faecium NM213 and Lactobacillus casei NM512 as novel probiotics with immunomodulatory properties. Microbiol. Immunol. 58 (10), 559–569. doi: 10.1111/1348-0421.12187 25130071

[B50] Montironi I. D. Moliva M. V. Campra N. A. Raviolo J. M. Bagnis G. Cariddi. L. N.. (2020). Characterization of an Enterococcus faecium strain in a murine mastitis model. J. Appl. Microbiol. 128 (5), 1289–1300. doi: 10.1111/jam.14554 31840319

[B51] Peng X. Wang R. Hu L. Zhou Q. Liu Y. Yang M.. (2019). Enterococcus faecium NCIMB 10415 administration improves the intestinal health and immunity in neonatal piglets infected by enterotoxigenic Escherichia coli K88. J. Anim. Sci. Biotechnol. 10 (1), 72. doi: 10.1186/s40104-019-0376-z 31452881 PMC6702752

[B52] Qin Y. Zhang L. L. Ye Y. R. Chen Y. T. Jiao Z. (2022). Parametric population pharmacokinetics of linezolid: A systematic review. Br. J. Clin. Pharmacol. 88 (9), 4043–4066. doi: 10.1111/bcp.15368 35484096

[B53] Rice L. B. Carias L. Rudin S. Vael C. Goossens H. Konstabel C.. (2003). A potential virulence gene, hylEfm, predominates in Enterococcus faecium of clinical origin. J. Infect. Dis. 187 (3), 508–512. doi: 10.1086/367711 12552437

[B54] Shi Y. Zhai M. Li J. Li B. (2020). Evaluation of safety and probiotic properties of a strain of Enterococcus faecium isolated from chicken bile. J. Food Sci. Technol. 57 (2), 578–587. doi: 10.1007/s13197-019-04089-7 32116367 PMC7016052

[B55] Soheili S. Ghafourian S. Sekawi Z. Neela V. Sadeghifard N. Ramli R.. (2014). Wide distribution of virulence genes among enterococcus faecium and enterococcus faecalis clinical isolates. Scientific World Journal. 2014, 623174. doi: 10.1155/2014/623174 25147855 PMC4124215

[B56] Thurlow L. R. Thomas V. C. Narayanan S. Olson S. Fleming S. D. Hancock L. E. (2010). Gelatinase contributes to the pathogenesis of endocarditis caused by Enterococcus faecalis. Infect. Immun. 78 (11), 4936–4943. doi: 10.1128/IAI.01118-09 20713628 PMC2976315

[B57] Tian F. S. Wang J. Yang Y. J. Liu C. Li C. J. Lv W. F. (2017). Isolation and screening of lactic acid bacteria from vagina of healthy dairy cows and their probiotic characteristics. J. Chin. Veterinary Med. 37 (03), 519–522. doi: 10.16303/j.cnki.1005-4545.2017.03.25

[B58] Torres C. Alonso C. A. Ruiz-Ripa L. León-Sampedro R. Del Campo R. Coqu,e T. M. (2018). Antimicrobial Resistance in Enterococcus spp. of animal origin. Microbiol. Spectr. 6 (4). doi: 10.1128/microbiolspec.ARBA-0032-2018 PMC11633606 30051804

[B59] Vandenesch F. Lina G. Henry T. (2012). Staphylococcus aureus hemolysins, bi-component leukocidins, and cytolytic peptides: a redundant arsenal of membrane-damaging virulence factors? Front. Cell Infect. Microbiol. 2. doi: 10.3389/fcimb.2012.00012 PMC3417661 22919604

[B60] Wang Y. Yang W. Zhang G. (2013). Effects of adding Enterococcus faecium in diet on growth performance, intestinal flora and immune function of weaned piglets. J. Anim. Nutr. 25 (05), 1069–1076. doi: 10.3969/j.issn.1006-267x.2013.05.023

[B61] Wang W. Cai H. Zhang A. Chen Z. Chang W. Liu G.. (2020). Enterococcus faecium modulates the gut microbiota of broilers and enhances phosphorus absorption and utilization. Anim. (Basel). 10 (7), 1232. doi: 10.3390/ani10071232 PMC7401662 32698425

[B62] Wu L. X. Huang W. X. Wang G. F. Sun X. P. (2009). Study on the pathogenicity of Enterococcus like virulence gene hyl. J. Biomed. Engineering. 26 (03), 601–605. doi: 10.3321/j.issn:1001-5515.2009.03.031 19634681

[B63] Xiao H. Zhang Y. Wang Y. R. Ni Y. Q. (2019). Analysis of drug resistance and virulence genes of Enterococcus faecium in raw milk and artisanal cheese in northwest Xinjiang. J. Microbiol. 39 (01), 37–43. doi: 10.3969/j.issn.1005-7021.2019.01.006

[B64] Xu Q. Y. Wang M. Z. Huo Y. Y. Luo Y. H. (2018). Effects of Enterococcus faecium preparation on serum biochemical, antioxidant and immune indexes of cows in the middle lactation period. Feed industry. 39 (19), 22–29. doi: 10.13302/j.cnki.fi.2018.19.005

[B65] Yang J. Wang H. Zhu W. T. Zhang G. Jin D. (2017). Antibiotic susceptibility and multilocus sequence typing of Enterococcus faecium strains isolated from foods and environment in Zigong, Sichuan. Dis. surveillance. 32 (12), 925–930. doi: 10.3784/j.issn.1003-9961.2017.12.008

[B66] Yu L. Peng Z. Dong L. Wang H. Shi S. (2019). Enterococcus faecium NCIMB 10415 supplementation improves the meat quality and antioxidant capacity of muscle of broilers. J. Anim. Physiol. Anim. Nutr. (Berl). 103 (4), 1099–1106. doi: 10.1111/jpn.13097 31025778

[B67] Yuksekdag Z. Ahlatcı N. S. Hajikhani R. Darilmaz D. O. Beyatli Y. (2021). Safety and metabolic characteristics of 17 Enterococcus faecium isolates. Arch. Microbiol. 203 (9), 5683–5694. doi: 10.1007/s00203-021-02536-8 34468805

[B68] Zhai M. K. (2017). Study on the probiotic properties and safety of a strain of Enterococcus faecium (Tai'an City, Shandong Province: University of Chicago Shandong Agricultural University).

[B69] Zhang M. Luo Q. Wei J. Liu Q. Luo P. (2021). Isolation and identification of bacteriocin-producing Enterococcus faecium and its antibacterial properties [J]. Food Science. 42 (06), 171–177. doi: 10.7506/spkx1002-6630-20191213-147

[B70] Zhang L. Y. Meng L. Z. Zhuan Q. R. Fu X. W. Hou Y. P. (2022). Analysis of intestinal flora changes in ICR mice after transmission based on microbial 16SrRNA sequencing technology[J]. J. China Agric. University. 27 (07), 126–136. doi: 10.11841/j.issn.1007-4333.2022.07.12

[B71] Zhang L. Yue H. Y. Zhang H. J. Xu L. Wu S. G. Yan H. J.. (2009). Transport stress in broilers:I. Blood metabolism, glycolytic potential, and meat quality. Poult. Sci. 88 (11), 2468. doi: 10.3382/ps.2009-00128 19762854

[B72] Zheng J. X. Bai B. Lin Z. W. Pu Z. Y. Yao W. M. Chen Z.. (2018). Characterization of biofilm formation by Enterococcus faecalis isolates derived from urinary tract infections in China. J. Med. Microbiol. 67 (1), 60–67. doi: 10.1099/jmm.0.000647 29148361 PMC5882073

[B73] Zhou B. Liu F. H. Cui D. F. Nie X. H. Wang X. T. Bai Y. (2011). Study on the production and characteristics of enterococcus faecium E9 [J]. Chin. J. Veterinary Med. 45 (02), 16–19. doi: 10.3969/j.issn.1002-1280.2011.02.005

